# Association of Yogurt and Dietary Supplements Containing Probiotic Consumption With All-Cause and Cause-Specific Mortality in US Adults: A Population-Based Cohort Study

**DOI:** 10.3389/fnut.2022.803076

**Published:** 2022-02-07

**Authors:** Ping Lin, Xuezhen Gui, Zongan Liang, Ting Wang

**Affiliations:** ^1^Department of Respiratory and Critical Care Medicine, West China School of Medicine and West China Hospital, Sichuan University, Chengdu, China; ^2^Division of Pulmonary Diseases, State Key Laboratory of Biotherapy of China, Sichuan University, Chengdu, China

**Keywords:** probiotic, yogurt, NHANES, mortality, population

## Abstract

**Background:**

Although probiotic intake had beneficial effects on several specific disorders, limited evidence was available about the benefits of probiotic intake in the general population. This study aimed to evaluate the relationship between yogurt (as a natural probiotic source) and dietary supplements containing probiotic consumption and mortality in US adults.

**Methods:**

We conducted an observational cohort study comprised of a nationally representative sample of adults who were enrolled in the National Health and Nutrition Examination Survey (NHANES) between 1999 and 2014. Individuals were linked to the US National Death Index.

**Results:**

We included 32,625 adults in our study. Of the study cohort, 3,539 participants had yogurt consumption, 213 had dietary supplements containing probiotic consumption, and the remaining participants (28,873) did not have yogurt and/or dietary supplements containing probiotic consumption. During 266,432 person-years of follow-up, 3,881 deaths from any cause were ascertained, of which 651 were due to cardiovascular disorders and 863 were due to cancer. Weighted Cox proportional hazards models suggested that yogurt consumption was inversely associated with all-cause mortality (adjusted hazard ratio (HR), 0.83 [95% confidence interval (CI), 0.71–0.98]) but not cardiovascular mortality (adjusted HR, 0.68 [95%CI, 0.43–1.08]) and cancer mortality (adjusted HR, 1.00 [95%CI, 0.72–1.38]). However, dietary supplements containing probiotic were not associated with decreased all-cause and cause-specific mortality.

**Conclusions:**

The present study suggested that yogurt consumption was associated with a lower risk of all-cause mortality among U.S. adults. Yogurt consumption in diet might be a sensible strategy for reducing the risk of death.

## Introduction

The human gastrointestinal tract harbors a complex community of microbes called the gut microbiota (1). The composition and metabolic activity of gut microbiota are now known to co-develop with the host beginning at birth and are under the influence of numerous factors (2). Gut microbiota and its disturbance have been associated with the pathogenesis of both intestinal and extra-intestinal disorders including gastroenterological diseases, metabolic disorders, cardiovascular diseases, autoimmune diseases, and neuropsychiatric disorders (3–7).

Probiotic intake might restore and maintain normal microbiota composition and function (8). Therefore, probiotic intake might have a positive impact on human health (9). Previous studies had suggested that probiotic might improve cognitive function and metabolic status (10), alleviate symptoms of various gastrointestinal disorders (11), decrease circulating levels of inflammatory biomarkers (12), improve glycemic and blood pressure control (13, 14), assist weight management in patients with type 2 diabetes (15), reduce antibiotic resistance (16), and prevent rhinovirus infections in preterm infants (17). Although published studies showed that probiotic intake had beneficial effects on people with specific disorders, limited evidence was available on the benefits of probiotic intake in the general population. Therefore, the recommendation to implement using of probiotic to provide health benefits in the general population needed further investigation (18).

In this study, we aimed to examine the relationship between yogurt (as a natural probiotic source) and dietary supplements containing probiotic consumption and mortality in a large cohort of participants in the National Health and Nutrition Examination Survey (NHANES), 1999–2014. We hypothesized that yogurt and dietary supplements containing probiotic consumption would be negatively associated with mortality.

## Methods

### Study Population

The NHANES survey is a national program of studies aimed to evaluate the health and nutritional status of the non-institutionalized US population using a complex, multistage, probability sampling design (19). The survey was conducted periodically since 1999 and a nationally representative sample of about 5,000 persons was examined each year. Data were collected by standardized in-person interviews and physical examinations.

**Figure 1:**
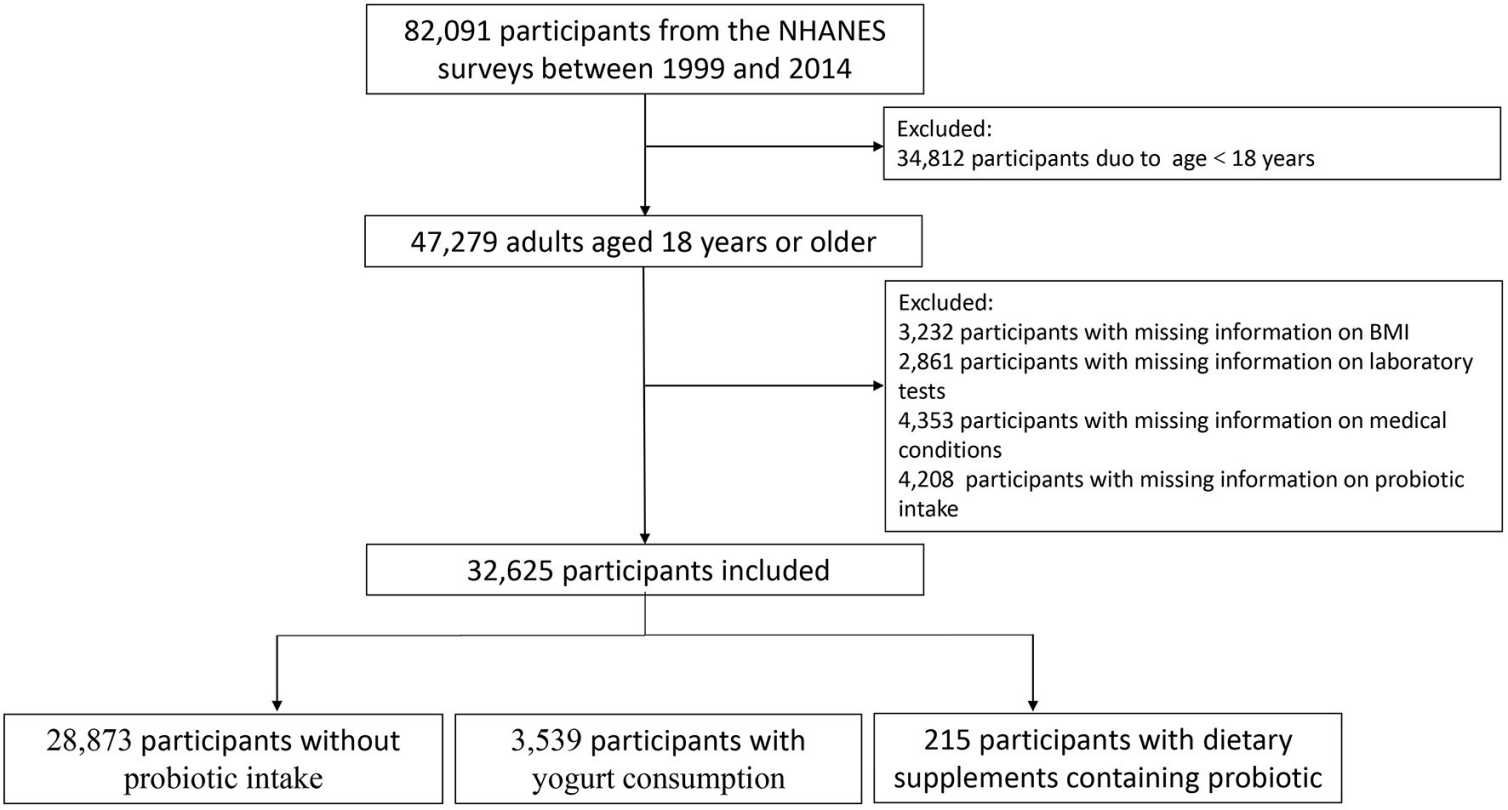
Flowchart of patient selection.

In the present study, 82,091 participants from the NHANES surveys between 1999 and 2014 were included. All participants were linked to the US National Death Index (NDI), which provided mortality follow-up data through December 31, 2015 (20). We excluded participants aged <18 years at interviews (*n* = 34,812) and those with missing information on BMI (*n* = 3,232), laboratory tests (white blood cell count, hemoglobin, platelet count, total bilirubin, creatinine, and blood urea nitrogen) (*n* = 2,861), medical conditions (hypertension, diabetes, asthma, congestive heart failure, coronary heart disease, stroke, chronic bronchitis, and cancer) (*n* = 4,353), yogurt and dietary supplements containing probiotic consumption (4,208). Therefore, a total of 32,625 participants remained in our cohort for analysis. Figure 1 presents a flowchart of participant selection.

### Assessments of Probiotic Intake

In this study, probiotic intake was considered when a participant reported consumption of yogurt or dietary supplements containing probiotics (21, 22). We utilized the Dietary Interview—First Day (the 24-h dietary recall interview before the survey) and Dietary Interview—Second Day (the second 24-h dietary recall interview collected by telephone 3 to 10 days after the first interview) to assess yogurt consumption. We utilized the Dietary Supplement Use 30-Day (1999–2014), a questionnaire that collected personal interview data on food supplement use during a 30-day period before the interview date, to assess dietary supplements containing probiotic. The detailed probiotic consumption information is described in Supplementary Material 1.

### Statistical Analysis

We analyzed the data using appropriate sampling weights (1/4^*^WTDR4YR for 1999–2002 and 1/8^*^WTDR2D for 2003–2014) to account for the complex survey design applied by the NHANES survey. We described baseline characteristics using percentages for categorical variables and means and standard deviations for continuous variables. We compared baseline characteristics using the Mantel–Haenszel χ^2^ test for categorical variables and the linear regression for continuous variables. We used weighted Cox proportional hazards models to calculate the hazard ratios (HRs) and corresponding 95% confidence intervals (CIs). Survey-weight adjusted multivariable Cox proportional model was adjusted for potential confounders that had been shown to be associated with mortality. Model 1 was not adjusted for any factors. Model 2 was adjusted for age, sex, race, and body mass index (BMI). Model 3 was adjusted for age, sex, race, BMI, white blood cell count, hemoglobin, platelet count, total bilirubin, creatinine, blood urea nitrogen, hypertension, diabetes, asthma congestive heart failure, coronary heart disease, stroke, chronic bronchitis, and cancer. Subgroup analyses were conducted by examining demographic characteristics, including age (<60 y, ≥60 y), sex (man, female), race/ethnicity (Mexican American, Other Hispanic, Non-Hispanic White, Non-Hispanic Black, Other Race), and BMI (kg/m^2^) (<18.5, 18.5 to 24.9, 25 to 29.9, ≥30). We used Stata version 14.0 (Stata Corp) and R version 3.6.3 (R Foundation for Statistical Computing) for statistical analysis and considered a two-tailed *p*-value less than 0.05 to be statistically significant.

## Results

### Participant Characteristics

A total of 32,625 adults aged 18 years or older were included in this analysis. Of the study cohort, 3,539 participants had yogurt consumption, 213 had dietary supplements containing probiotic consumption, and the remaining participants (28,873) did not have yogurt and/or dietary supplements containing probiotic consumption. Characteristics of participants with different statuses of probiotic consumption were significantly different. Participants with yogurt or dietary supplements containing probiotic consumption were more likely to be older, to be a woman, to have low BMI, to be Non-Hispanic White, to have less hypertension, and to have cancer. The detailed baseline characteristics are shown in Table 1.

**Table 1:** Participants baseline demographic and clinical characteristics.

**Characteristic**	**Without probiotic intake (*N* = 32,965)**	**Yogurt consumption (*N* = 3,539)**	**Dietary supplements containing probiotic (*N* = 213)**	***p*-value**
**Baseline characteristics**				
Age (y)	46.2 ± 16.8	48.3 ± 16.4	50.1 ± 15.7	<0.001
Man (%)	49.7	35.4	27.3	<0.001
BMI (kg/m^2^)	28.6 ± 6.6	27.7 ± 6.4	27.1 ± 6.5	<0.001
Race (%)				<0.001
Mexican American	8.4	6.0	2.6	
Other Hispanic	5.1	4.2	5.2	
Non-Hispanic White	69.3	79.0	81.7	
Non-Hispanic Black	11.6	4.8	6.5	
Others	5.5	6.0	3.9	
**Laboratory tests**				
Hemoglobin (g/dL)	14.4 ± 1.5	14.1 ± 1.3	14.2 ± 1.1	<0.001
WBC (1,000 cells/uL)	7.3 ± 2.3	7.1 ± 2.4	6.8 ± 1.8	<0.001
Platelets (1,000 cells/uL)	258.5 ± 67.2	252.5 ± 65.4	249.0 ± 63.7	<0.001
Total bilirubin (umol/L)	12.2 ± 5.3	12.5 ± 5.1	12.8 ± 4.8	0.0012
Creatinine (umol/L)	78.1 ± 33.2	75.2 ± 20.4	75.1 ± 16.8	<0.001
BUN (mmol/L)	4.7 ± 1.9	4.8 ± 1.7	4.7 ± 1.6	0.0190
**Comorbidities**				
Hypertension (%)	29.7	26.8	24.7	0.0002
Diabetes mellitus (%)	8.1	6.4	9.9	0.0002
CHD (%)	3.4	3.1	2.8	0.5734
CHF (%)	2.3	1.8	1.5	0.0707
Asthma (%)	13.4	12.5	18.7	0.0070
Stroke (%)	2.6	1.7	2.3	0.0014
Chronic bronchitis (%)	6.3	4.1	9.4	<0.001
Cancer (%)	8.7	10.8	16.4	<0.001

*BMI, body mass index; WBC, white blood cell; BUN, blood urea nitrogen; CAD, coronary heart disease; CHF, congestive heart failure*.

*Continuous variables were expressed as means and standard deviations and categorical variables were expressed as percentages. Means and percentages are weighted*.

### Associations Between Probiotic Exposure and Mortality

During 266,432 person-years of follow-up, 3,881 deaths from any cause were ascertained, of which 651 were due to cardiovascular disorders and 863 were due to cancer. We evaluated associations of probiotic intake with mortality by logistic regression models with unadjusted and adjusted covariates (Table 2).

**Table 2:** Association of probiotic exposure with mortality in 32,625 participants of national health and nutrition examination survey, 1999–2014.

**Exposure**	**Model 1^a^ HR (95% CI)**	***p*-value**	**Model 2^b^ HR (95% CI)**	***p*-value**	**Model 3^c^ HR (95% CI)**	***p*-value**
**All-cause mortality**	
Yogurt	0.86 (0.73, 1.02)	0.096	0.79 (0.67, 0.93)	0.005	0.83 (0.71, 0.98)	0.035
Probiotic supplements	0.64 (0.36, 1.14)	0.132	0.67 (0.38, 1.19)	0.181	0.74 (0.43, 1.29)	0.300
**Cardiovascular mortality**	
Yogurt	0.66 (0.41, 1.04)	0.076	0.62 (0.39, 0.99)	0.048	0.68 (0.43, 1.08)	0.109
Probiotic supplements	0.83 (0.25, 2.72)	0.763	0.99 (0.30, 3.24)	0.993	1.13 (0.34, 3.67)	0.837
**Cancer mortality**						
Yogurt	1.03 (0.75, 1.41)	0.845	0.98 (0.71, 1.35)	0.904	1.00 (0.72, 1.38)	0.972
Probiotic supplements	1.12 (0.41, 3.07)	0.815	1.21 (0.45, 3.27)	0.699	1.34 (0.50, 3.62)	0.555

a *Unadjusted*.

b *Adjusted for age, sex, race, and body mass index*.

c *Adjusted for age, sex, race, body mass index, white blood cell count, hemoglobin, platelet count, total bilirubin, creatinine, blood urea nitrogen, hypertension, diabetes, asthma congestive heart failure, coronary heart disease, stroke, chronic bronchitis, and cancer*.

For unadjusted analyses (**Model 1**), yogurt consumption was not associated with a reduced risk of all-cause mortality (HR, 0.86 [95%CI, 0.73–1.02]), cardiovascular mortality (HR, 0.66[95%CI, 0.41–1.04]), and cancer mortality (HR, 1.03 [95%CI, 0.75–1.41]). After adjusting for potential confounders (**Model 2 and 3**), yogurt consumption was inversely associated with all-cause mortality (HR, 0.83 [95%CI, 0.71–0.98]) but not cardiovascular mortality (HR, 0.68 [95%CI, 0.43–1.08]) and cancer mortality (HR, 1.00 [95%CI, 0.72–1.38]) (**Model 3**).

However, dietary supplements containing probiotic were not associated with decreased all-cause and cause-specific mortality (Table 2). The unadjusted RRs of all-cause, cardiovascular, and cancer mortality were 0.64 (95% CI 0.36, 1.14), 0.83 (95% CI 0.25, 2.72), 1.12 (95% CI 0.41, 3.07), respectively. The adjusted RRs of all-cause, cardiovascular, and cancer mortality were 0.74 (95% CI 0.43, 1.29), 1.13 (95% CI 0.34, 3.67), 1.34 (95% CI 0.50, 3.62), respectively.

### Subgroup Analysis

We further explored the association of yogurt consumption with all-cause mortality according to gender, age, race/ethnicity, and BMI (Table 3). We found that the association between yogurt consumption and decreased all-cause mortality was statistically significant among female, older participants (aged ≥ 60 y), and Non-Hispanic Black, while the association was weak and not statistically significant in other gender, ages, race/ethnicity, and weight subgroups.

**Table 3:** Association of yogurt consumption with mortality according to age, sex, race/ethnicity, and BMI.

**Subgroup**	All-cause mortality HR (95% CI)^*^	***p*-value**
**Gender**		
Male	0.89 (0.70, 1.15)	0.403
Female	0.77 (0.62, 0.97)	0.028
**Age**		
Aged <60 y	0.96 (0.65, 1.40)	0.851
Aged ≥ 60 y	0.80 (0.68, 0.96)	0.017
**Race/Ethnicity**		
Mexican American	0.66 (0.40, 1.10)	0.113
Other Hispanic	1.85 (0.94, 3.64)	0.073
Non-Hispanic White	0.87 (0.73, 1.05)	0.170
Non-Hispanic Black	0.54 (0.30, 0.97)	0.042
Other Race	0.40 (0.13, 1.21)	0.107
**Weight**		
Underweight (BMI <18.5)	0.44 (0.16, 1.18)	0.104
Normal (BMI, 18.5 to 24.9)	0.78 (0.59, 1.04)	0.096
Overweight (BMI, 25 to 29.9)	0.86 (0.66, 1.14)	0.313
Obese (BMI ≥ 30)	0.83 (0.60, 1.15)	0.275

* *Adjusted for age, sex, race, body mass index, white blood cell count, hemoglobin, platelet count, total bilirubin, creatinine, blood urea nitrogen, hypertension, diabetes, asthma congestive heart failure, coronary heart disease, stroke, chronic bronchitis, and cancer*.

## Discussion

In this large, nationally representative sample of US adults, we observed that participants with yogurt consumption had a lower risk for all-cause mortality compared with participants without yogurt consumption that persisted after adjustment for demographic characteristics, laboratory tests, and clinic comorbidities. In subsequent subgroup analyses, this yogurt–mortality inverse association was found to be present predominantly among female, older participants, and Non-Hispanic Black.

The association between yogurt consumption and mortality was inconsistent in previous studies. Schmid et al. reported that yogurt consumption was associated with a decreased risk of all-cause mortality (HR, 0.89 [95%CI, 0.86–0.93]) in the Nurses' Health Study and The Health Professionals Follow-Up Study, whereas Soedamah-Muthu et al. and Praagman et al. failed to discover a significant association between yogurt consumption and all-cause mortality in the Whitehall II cohort (HR, 0.74 [95%CI, 0.53–1.05]) and the European Prospective Investigation into Cancer and Nutrition-Netherlands cohort (HR, 0.95 [95%CI, 0.85–1.07]) (23, 24). The inconsistent findings might be due to different sociodemographic characteristics and follow-up times. In the present analysis, we observed that individuals with yogurt consumption had a 17% lower risk for all-cause mortality. This study was a national survey with a large sample size and a long follow-up (mean 8.1 years), which could offer reliable evidence and further reinforce earlier results. Moreover, we further conducted subgroup analyses by examining demographic characteristics (age, sex, race/ethnicity, and BMI). We found that the health benefits of yogurt consumption were particular among female, older participants (aged ≥ 60 y), and Non-Hispanic Black, indicating that these individuals were more likely to benefit from yogurt consumption. Unfortunately, the exact reasons for these observed demographic characteristics differences in the inverse associations were not clear. Sex-related differences might be due to the metabolism of sex hormones regulated by the gut microbiome (25).

We observed a reduction in mortality in yogurt consumption rather than dietary supplements containing probiotic in this study. Yogurt is a nutrient-dense fermented dairy product and is associated with high nutritional value (26). In addition to probiotic strains, yogurt is also a good source of abundant micronutrients and macronutrients, including calcium, Fe, Mn, zinc, vitamins B-6/B-12, riboflavin, and protein (27, 28). These nutrients are essential for the sustenance of the functioning of the human body. Therefore, these nutrients might have a role in the beneficial effects of yogurt consumption compared to the supplementation of probiotic strains into a carrier liquid only. In addition, only a small number of people (about 0.65%) who had a consumption of dietary supplements containing probiotic in diet might be responsible, at least in part, for these unexpected results.

Our results were supported by the existence of data showing a biological plausibility for the health benefits of probiotic. First, probiotic intake might be helpful to reduce risk factors for all-cause mortality and cardiovascular mortality such as obesity, hypertension, and type 2 diabetes (29–31). Second, probiotic foods had been reported to repress oxidative stress and inflammatory marker profile in both healthy persons and patients with disorders (32–34). Third, data also suggested that the use of probiotic might improve endothelial function and reduce arterial stiffness (35). Fourth, previous investigators found that probiotic intake might reduce enteric bacterial translocation and suppress the growth of pathogenic microbiota, indicating that probiotic could prevent enteric infections and inhibit the growth of intestinal carcinoma (36, 37). Furthermore, intake of probiotic significantly might improve vitamins and minerals status such as vitamin K and calcium which played an important role in maintaining normal physiological activities (38).

An important implication from this study was that yogurt consumption might be an important public health strategy that had been associated with decreased mortality risk among adults. Our study revealed that only 11.4% of participants had exposure to yogurt and/or dietary supplements containing probiotic among U.S. adults, which were similar to those previously reported (13.1%) (39). It indicated that the consumption of probiotic food remained relatively unpopular and there was still much potential for improvement in US adults. Appropriate strategies were needed to encourage the wider population to accept probiotic food such as health promotion and health education around the benefits of probiotic food.

This study had several strengths. The use of a large size, nationally representative sample would improve the ability to address the impact of possible confounding and increase the generalizability and external validity of our results. In addition, we further conducted subgroup analyses according to age, gender, race/ethnicity, and BMI, and found that female, older participants (aged ≥ 60 y), and Non-Hispanic Black were more likely to benefit from yogurt consumption. At the same time, several limitations of this study should be recognized. First, dietary data was self-reported in the NHANES survey, which might result in measurement error inevitably. Second, due to the lack of detailed information about yogurt consumption, we could not conduct subgroup analyses to explore the dose-response association between yogurt consumption and mortality. Third, this was a retrospective, observational study, which was subject to confounding bias. Although we carefully controlled for possible confounding factors, residual confounding or unmeasured confounding could not be fully ruled out. Moreover, because dietary data was collected based on the Dietary Interview-First Day/Second Day and Dietary Supplement Use 30-Day, dietary changes could not be excluded after the survey. However, dietary changes were likely to lead to an underestimation instead of an overestimation of probiotic benefits.

## Conclusions

In this study, we found that yogurt consumption was associated with a lower risk of all-cause mortality in the general population, especially among female, older participants, and Non-Hispanic Black. Yogurt consumption in diet might be a sensible strategy for reducing the risk of death.

## Data Availability Statement

Publicly available datasets were analyzed in this study. Data used for this study are available on the NHANES website: https://wwwn.cdc.gov/nchs/nhanes/.

## Ethics Statement

The studies involving human participants were reviewed and approved by the institutional review board of the National Center for Health Statistics, CDC. The patients/participants provided their written informed consent to participate in this study.

## Author Contributions

PL and XG contributed to study design, data collection, data analysis, and drafting the article. ZL and TW contributed to study design, critical revision and submitted the report for publication. All authors read and approved the final manuscript.

## Funding

This study was funded by the National Key Research and Development Program of China (Grant No. 2016YFC1304303) and the National Natural Science Foundation of China (Grant No. 31701011).

## Conflict of Interest

The authors declare that the research was conducted in the absence of any commercial or financial relationships that could be construed as a potential conflict of interest.

## Publisher's Note

All claims expressed in this article are solely those of the authors and do not necessarily represent those of their affiliated organizations, or those of the publisher, the editors and the reviewers. Any product that may be evaluated in this article, or claim that may be made by its manufacturer, is not guaranteed or endorsed by the publisher.

## Abbreviations

NHANES: the National Health and Nutrition Examination Survey
NDI: the National Death Index
HR: hazard ratio
CI: confidence interval
BMI: body mass index.
